# Modeling epithelial-mesenchymal transition in patient-derived breast cancer organoids

**DOI:** 10.3389/fonc.2024.1470379

**Published:** 2024-10-14

**Authors:** Neta Bar-Hai, Rakefet Ben-Yishay, Sheli Arbili-Yarhi, Naama Herman, Vered Avidan-Noy, Tehillah Menes, Aiham Mansour, Fahim Awwad, Nora Balint-Lahat, Gil Goldinger, Goni Hout-Siloni, Mohammad Adileh, Raanan Berger, Dana Ishay-Ronen

**Affiliations:** ^1^ Oncology Institute, Shaba Medical Center, Ramat-Gan, Israel; ^2^ Faculty of Medicine, Tel Aviv University, Tel Aviv, Israel; ^3^ Department of General Surgery, Shaba Medical Center, Ramat-Gan, Israel; ^4^ Institute of Pathology, Shaba Medical Center, Ramat-Gan, Israel; ^5^ Department of Surgery, Mount Scopus, Hadassah Hebrew University Medical Center, Jerusalem, Israel

**Keywords:** breast cancer, EMT, cell plasticity, tumor heterogeneity, organoids

## Abstract

Cellular plasticity is enhanced by dedifferentiation processes such as epithelial-mesenchymal transition (EMT). The dynamic and transient nature of EMT-like processes challenges the investigation of cell plasticity in patient-derived breast cancer models. Here, we utilized patient-derived organoids (PDOs) as a model to study the susceptibility of primary breast cancer cells to EMT. Upon induction with TGF-β, PDOs exhibited EMT-like features, including morphological changes, E-cadherin downregulation and cytoskeletal reorganization, leading to an invasive phenotype. Image analysis and the integration of deep learning algorithms enabled the implantation of microscopy-based quantifications demonstrating repetitive results between organoid lines from different breast cancer patients. Interestingly, epithelial plasticity was also expressed in terms of alterations in luminal and myoepithelial distribution upon TGF-β induction. The effective modeling of dynamic processes such as EMT in organoids and their characteristic spatial diversity highlight their potential to advance research on cancer cell plasticity in cancer patients.

## 1 Introduction

Breast cancer ranks as a leading cause of cancer-related deaths among women, primarily attributed to its heterogeneity and propensity to metastasize and evade treatments (1–4).

The process of cancer cell invasion and dissemination encompasses several fundamental steps: local invasion, intravasation, bloodstream survival, extravasation, colonization, and evasion of therapeutic interventions. Each step demands different characteristics from the cancer cell, adding to the complexity and difficulty in understanding and combating this process. However, a common requirement for all these steps is cellular plasticity. Biological plasticity is defined as the ability to adapt and survive under variable circumstances. Cellular plasticity allows cells to accumulate and thrive in changing environments. Enhanced plasticity enables cancer cells to adapt to and survive in various hostile conditions and treatments, promoting persistence and progression (5–7). Understanding the mechanisms underlying cellular plasticity could open doors to improving survival and enhancing the quality of life of cancer patients (8). We previously demonstrated how cellular plasticity can be utilized to trans-differentiate cancer cells into post-mitotic, non-dividing adipocytes (9). Thus, suggesting how comprehending cancer cell plasticity may lead to novel therapeutic approaches.

During EMT, a plethora of phenotypic and functional cellular changes enhance cancer plasticity (10–13). The dedifferentiation process typical to EMT includes the downregulation of adhesion proteins such as E-Cadherin and the change in cell polarity triggered by the rearrangement of the cytoskeleton (14). EMT was originally described in developmental biology, when during embryogenesis cell migration enabled the formation of the three germ layers (15, 16). Cancer invasion, metastasis formation, and drug resistance are all significantly impacted by pathological reactivation of the EMT process (7, 17–19). Over the past decade, the field of EMT has witnessed significant advancements. Nonetheless, as a transient and dynamic phenomenon, it presents substantial challenges for accurate modeling (20). The utilization of preclinical genetic mouse models, including both knock-out and knock-in techniques targeting EMT transcription factors (21–25) has been instrumental in enhancing our understanding of EMT. Lineage tracing models based on the activation and/or switching of fluorescent reporters driving EMT, particularly those focused on breast (26–28), pancreatic (29) and colorectal cancers (30) have further elucidated the complexities of this process. Approaching the clinical application of EMT modeling, Soundararajan et al.’s study provides a notable example of a clinically relevant mouse model. Their research shows that a gene expression signature from mouse embryos, indicative of high cellular plasticity, can predict breast cancer metastasis (31). As for EMT assessment in patient-derived assays, detection of EMT markers in tumor samples is widely used (32, 33). In fact, cells that have undergone EMT are not frequently observed in tumor samples, presumably because biopsies represent a precise moment during tumor development, whereas cells undergoing EMT could be switching between hybrid epithelial/mesenchymal states. A recently published methodology suggests an interpretable, scalable, machine learning-based method to assess EMT status directly from Hematoxylin and Eosin (H&E)-stained images, which may improve traditional biopsy analysis (34). A unique approach involves prospectively collecting surgical specimens from primary tumors, adjacent normal tissue, and metastatic sites. This method aims to capture the dynamic processes characterized by high plasticity, potentially facilitating a deeper understanding of EMT dynamics in tumor progression (35).

PDOs may offer a promising complementary approach to study cancer cells susceptibility to EMT in human tissues. Owing to their complex heterocellular architecture and functionality, PDOs are particularly suited to study evolving, transitory, and heterogeneous processes (36–40). While spheroid cultures are typically derived from a single cell clone and thus exhibit high homogeneity, patient-derived organoids offer a more accurate representation of the complexities and heterogeneity inherent in human tumors. Thus, PDOs propose a model for studying cancer biology and treatment responses, enhancing their relevance to patient outcomes and improving their translatability to clinical settings (41).

While PDO cultures recapitulate original tissue architecture and heterogeneity, the culture conditions essential for the proper growth of PDOs interfere with dedifferentiation processes. Specifically, a potent inducer of EMT *in vitro* and *in vivo* is the cytokine TGF-β. Yet, organoids culture medium contains various compounds inhibiting TGF-β signaling to maintain epithelial organization. Optimizing PDO culture medium for TGF-β induction reveals the possibility to induce cancer cell plasticity in cancer organoids.

Here, we demonstrate the potential of EMT modeling in breast cancer PDOs utilizing microscopy and histology-based approaches to highlight inter- and intra-organoid variability. Through the manifestation of EMT-like and invasive phenotypes under varied durations of TGF-β induction, we demonstrate that PDOs proficiently capture dynamic cellular processes as cell plasticity and invasiveness. This approach addresses the limitations of existing research models, which may miss critical aspects of EMT, including its dynamic nature, heterogeneity, and spatial context in patient-derived cultures. Recognizing that PDO expansion can be challenging in primary cultures, our study aimed to develop a robust methodology for evaluating EMT susceptibility in early PDO cultures. Therefore, we focused on image analysis based primarily on confocal microscopy to evaluate changes typical for EMT; changes in organoids morphology, cytoskeleton reorganization, E-Cadherin downregulation leading to an invasive migratory phenotype. Utilizing deep learning algorithms we established EMT-quantification tools optimized for PDO models. Applied across diverse breast tumors, this work underscores the substantial potential of PDOs to advance research on cancer cell plasticity and personalized medicine.

## 2 Materials and methods

### 2.1 Cell lines

Murine epithelial tumor cells derived from a mammary tumor of an MMTV-PyMT transgenic mouse (MMTV: promoter, PyMT: polyoma middle T-antigen oncogene) as previously described (42). Cells were cultured in high glucose Dulbecco’s modified eagle medium (DMEM (Sartorius; 01-055-1A)) supplemented with 10% Fetal Bovine Serum (FBSS; ThermoFisher; A5256701), x1 Penicillin/streptomycin (Biowest; L0022-100) and 2mM L-Glutamine (Biowest; X0550-100). All cell lines were grown at 37°C, 5% CO2, 95% humidity. TGF-β (R&D Systems; 240-B); instead of, was added to the growth medium at a concentration of 2ng/ml, with medium changes occurring every 3 days.

### 2.2 Breast cancer organoid culture

For the establishment of patient-derived tumor breast organoids, tumor breast tissues were obtained via the Sheba Tissue Bank from patients that underwent mastectomy or lumpectomy under informed consent. Following published protocol (43), the tissue was both mechanically and enzymatically digested, and isolated cells were plated in adherent Cultrex growth-factor-reduced basement membrane extract (BME) type 2 drops (R&D Systems; 3533-005-02). Organoids were overlaid with organoid culture medium containing Advanced DMEM DMEM/F12 (Thermofisher; 12634010), supplemented with 10mM HEPES (GIBCOTM; 15630-080), 2mM GlutaMAX (GIBCOTM; 35050-061), x1 Penicillin streptomycin (Biowest;L0022-100), 10% R-spondin-1-conditioned medium (RCM) produced from HEK293 HA–Rspo1–Fc cells (Cultrex^®^ HA–R-spondin-1–Fc 293T cells; 3710-001-01), 10% Noggin-conditioned medium (NCM) produced from HEK293 cells stably transfected with pcDNA3-mouse NEO insert (to confer neomycin resistance; cells for NCM production were kindly provided by the Hubrecht Institute, Utrecht, The Netherlands), x1 B27 (ThermoFisher; 17504044)), 100 ng/mL A83-01 (Tocris Bioscience; 2939), 50 ng/mL EGF (PeproTech; AF-100-15). When organoids were first established or expended, 10 mM Y-27632 (ROCK inhibitor; Sigma Aldrich; Y0503) was added to the medium. Medium was changed every 4 days, and organoids were passaged every few weeks using mechanical shearing with TrypLE Express (Invitrogen; 12605036). Use of human tissues was approved by the local ethics committee and by the Associate Director at the Sheba Medical Center (approval no. 7188-20-smc) and informed consent was obtained from all tissue donors. Investigations were conducted according to the principles expressed in the Declaration of Helsinki.

### 2.3 Histological characterization

#### 2.3.1 Tissues

Tissues were processed according to standard pathological procedures. Tissues were fixed in buffered formalin, embedded in paraffin, and sectioned using a microtome. Sections were then placed on histologic slides and stained using H&E. Slides were scanned at x40 magnification using the Philips IntelliSite Ultra-Fast scanner (Philips Digital Pathology Solutions, Best, Netherlands).

#### 2.3.2 Organoids

Following treatments, the organoids were extracted from BME by 1h incubation with Cell Recovery Solution (Corning^®^; 354253) on ice for 1 hour and fixed in 4% paraformaldehyde for 30 minutes. After fixation, the organoids were washed with PBS, suspended in 1% Agarose and embedded in paraffin to create formalin-fixed paraffin-embedded (FFPE) blocks. FFPE blocks were sectioned using a microtome. Slides were then scanned at x40 magnification using VENTANA^®^ DP 200 slide scanner (Roche, Basel, Switzerland).

### 2.4 EMT induction in patient derived organoids

Whole organoids were suspended in BME, plated in an 18-well μ Slide (ibidi; 81816) and covered with the appropriate growth medium for the duration of the experiment (3-10 days). Control group organoids were covered with organoid culture medium, while TGF-β group organoids were covered with EMT-optimized medium (EMT medium): organoid culture medium without A-8301, supplemented with TGF-β. Medium was changed every 3 days.

### 2.5 Immunofluorescence staining

Following treatments, the organoids were fixed in 4% paraformaldehyde for 30 minutes, permeabilized with 0.3% Triton X100 in phosphate buffered saline (PBS) for 30 min, then blocked with 3% Bovine serum albumin (BSA; Avantor; 0332-50G), in PBST (0.01% Triton X-100 in PBS) for 1 hour. The organoids were incubated with a primary antibody (Rabbit anti E-Cad (cell signaling; #3195), 1:200, or anti-Cytokeratin 14 (Ck14) conjugated to Alexa Fluor 647 ((abcam; ab206100),1:100) or anti-Cytokeratin 8 (Ck8) conjugated to Alexa Fluor 488 (abcam; ab192467; 1:100)) for 2 hours at room temperature (RT), then for 1 hour at RT with a secondary antibody Alexa Fluor 647 labeled gout anti Rabbit (abcam; ab150083), 1:500) together with Phalloidin-iFluor 488 Reagent ((abcam; ab176753), 1:1000). Antibodies were diluted in 3% BSA in PBST, and incubations were followed by three washes of 5 min with PBST. DNA staining was performed with 4′,6-diamidino-2-phenylindole (DAPI; D1306; Invitrogen), for 10 min, and then organoids were covered with PBS.

### 2.6 Confocal microscopy

Confocal imaging was performed on a confocal LSM700 ZEISS microscope, using a 40× oil lens, NA 1.518 and on TCS SP8 Leica Confocal microscope, Leica Microsystems.

### 2.7 Image processing and analysis

#### 2.7.1 E-Cadherin mean intensity measurements

To isolate individual cells within an organoid image taken by confocal microscopy, we utilized CellPose3, a deep neural network-based instance segmentation algorithm (44). The algorithm supports designating different channels as the nucleus and cytoplasm in order to facilitate better segmentation. Here, DAPI was used as the nucleus marker and Phalloidin as the cytoplasm marker. For each segmented cell, we obtained the segmentation mask and computed the mean gray level value (between 0 and 255) of the E-Cadherin channel on the activated mask pixels.

#### 2.7.2 Relative well-bottom adhesion area measurements

The well-bottom adhesion area was manually outlined and measured using the Fiji processing software package of ImageJ. The relative adhesion area was calculated as the ratio of the well-bottom adhesion area to the total image area.

### 2.8 Biostatistics

#### 2.8.1 E-Cadherin intensity

E-Cadherin intensity was assessed in patient-derived organoids BR73T (n = 386 measurements) and BR83T (n = 50 measurements). To evaluate the differences between control and TGF-β treated PDOs, an unpaired t-test was employed. Data are expressed as mean ± SEM. Significance was determined with a p-value of ****<0.0001.

#### 2.8.2 Comparison of cell counts: manual vs. CellPose segmentation

To evaluate the differences between manual and CellPose segmentation, cell counts of randomly selected images were compared and paired t-test was employed. The analysis included 6 measurements per group, with non-significant p-value for control images and p-value of 0.018 for TGF-β images.

#### 2.8.3 Adhesion area

The relative well-bottom adhesion area was analyzed using unpaired t-tests for statistical comparisons between groups. The analysis included 18 measurements per group, with a p- with a p-value of 0.0034.

All statistical analyses were performed using GraphPad Prism software.

## 3 Results

### 3.1 Optimization of culture medium for EMT induction

**Figure 1:**
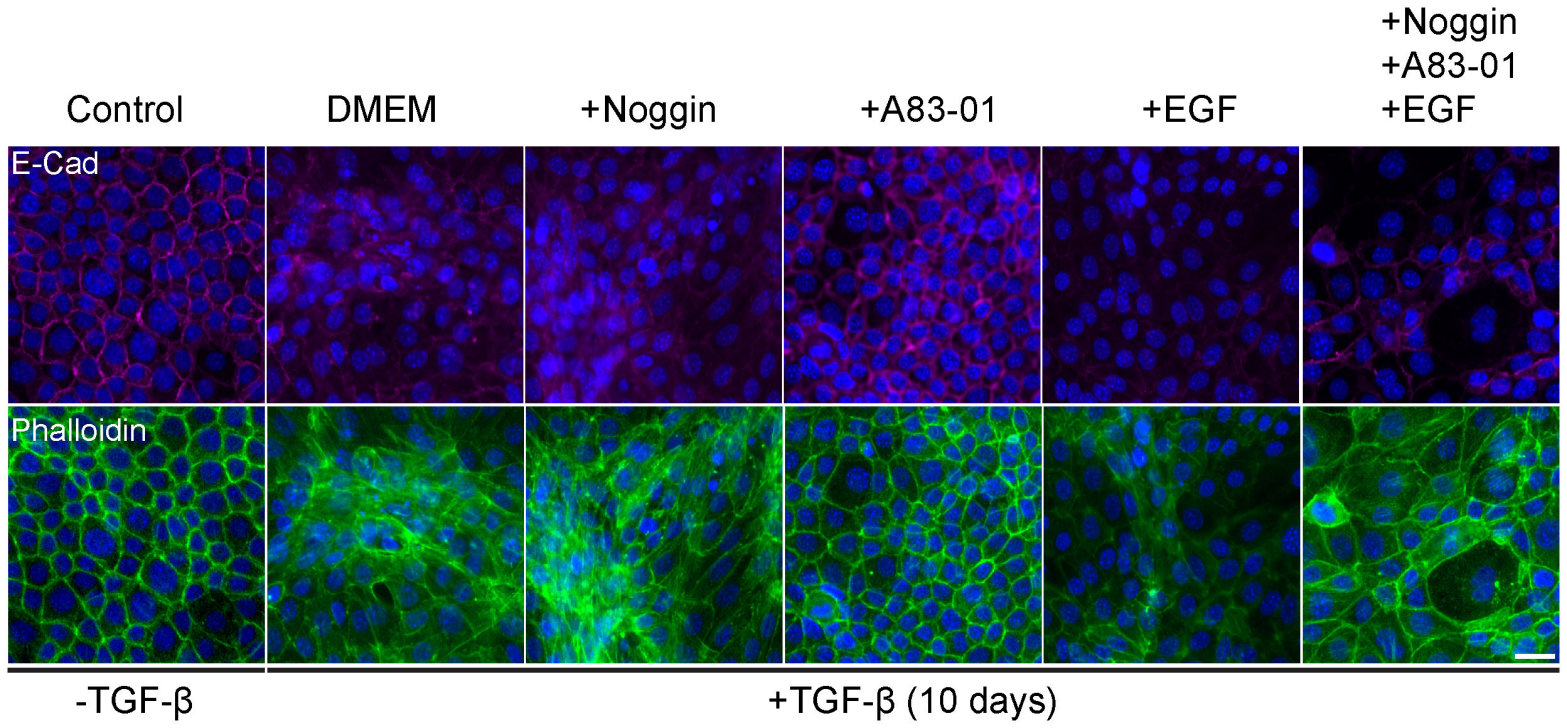
Organoids culture medium components interfere with EMT in murine breast cancer cells. Py2T cells were treated with TGF-β (2ng/ml) for 10 days to induce EMT, and DMEM culture medium was supplemented with various potential EMT regulators: Noggin (10%), A83-01 (500nM), EGF (5ng/ml). Cells were immunolabeled with antibody for the epithelial marker E-Cadherin (magenta, top) and counter-stained with DAPI to label cell nuclei (blue), and Phalloidin to label F-actin (green, bottom). Bar = 100µm.

TGF-β induced epithelial response is counteracted in organoid culture media by various compounds to maintain epithelial organization. Therefore, optimization of the culture medium was necessary to allow EMT induction with TGF-β exposure. For this purpose, we induced EMT in 2D cell cultures of Py2T murine breast cancer cells as previously described (42). The culture medium was supplemented with cytokines and growth factors from the organoid culture medium that were expected to interfere with TGF-β signaling. Specifically we used Noggin, an antagonist of bone morphogenetic proteins (BMPs) from the TGF-β-superfamily (45); A83-01, an inhibitor of TGF-β type I receptor ALK-5 (46); and epidermal growth factor (EGF) that has a known cross-talk with TGF-β signaling pathway via MAPK with common targets (47, 48). EMT was induced using TGF- β and was evaluated by visualizing actin fibers and E-cadherin expression (**Figure 1**). The results revealed that while Noggin seemed to enhance EMT in organoid cultures, A83-01 repressed EMT response and maintained epithelial characteristics upon TGF- β induction. Therefore, to enable proper EMT induction with TGF-β in PDOs, A83-01 was removed from the culture medium during EMT induction.

### 3.2 Modeling EMT in patient-derived breast cancer organoids

#### 3.2.1 PDOs recapitulate breast cancer architecture and demonstrate EMT-driven morphological changes

Breast cancer tissues were obtained from primary tumors of patients diagnosed with early-stage breast cancer undergoing surgical lumpectomy or mastectomy. No neoadjuvant treatment was administered to any of the patients prior to surgical resection of the primary tumor. The majority of the PDOs were established from tumor tissues with similar clinicopathological characteristics as described in **Table 1**. Namely, most of the organoids were established from intra-ductal carcinoma (IDC), hormone receptors positive (HR+), with Oncotype recurrence scores (RS) between 12-18, and derived from premenopausal patients. An exception is PDO BR19T, which was established from mucinous carcinoma in a postmenopausal patient. PDO BR73T is also noteworthy for being triple-positive IDC (HR+ and HER2-positive). It is worth mentioning that all organoid lines were derived from tumors that were either multifocal or multicentric. This was not an intentional selection criterion but rather a consequence of the necessity to obtain adequate tissue for both pathological analysis and organoids establishment from residual tissue in early breast cancer.

**Table 1:** Clinicopathological and organoid characteristics of patient derived organoids.

Organoid line	Patient			Tumor										
	Age (years)	Menopause	Comorbidities	Histology	Size (cm)	Staging			Receptors			Grade	Ki67 (%)	Oncotype RS
						T	N	M	ER (%)	PR (%)	HER2			
**BR19T**	56	Post	X	Multifocal Mucinous Carcinoma	4	2	0	0	100	40	–	2	15	21
**BR63T**	44	Pre	Asthma	Multifocal IDC	3.5	2	1	0	100	100	–	2	15	18
**BR72T**	47	Pre	Smoking	Multicentric IDC	4.4	2	0	0	100	100	–	2	20	11
**BR73T**	48	Pre	Hypothyroidism	Multifocal IDC	0.8	1	0	0	100	100	+	2	20	N/A
**BR83T**	42	Pre	X	Multifocal IDC	2.5	2	0	0	90	100	–	1	15	12

RS, Recurrence Score; IDC, Intraductal Carcinoma; N/A, Not Applicable.

Following establishment, triple-positive PDOs were fixed, embedded in paraffin to create FFPE blocks, and sectioned onto slides for H&E staining. Pathological evaluation of the H&E staining of both the original tissue and PDOs demonstrated that PDOs maintained tumor characteristics and faithfully recapitulated the histological and cytological profiles of the original tumor tissue (**Supplementary Figure 1**). Subsequent exposure of PDOs to TGF-β by culturing the organoids in EMT-medium (organoid culture medium without A-8301, supplemented with TGF-β) for 10 days revealed noticeable changes in overall morphology of the PDOs upon TGF-β induction compared to control, as illustrated by histological sections.

#### 3.2.2 Breast cancer PDOs undergo gradual EMT upon TGF-β exposure

**Figure 2:**
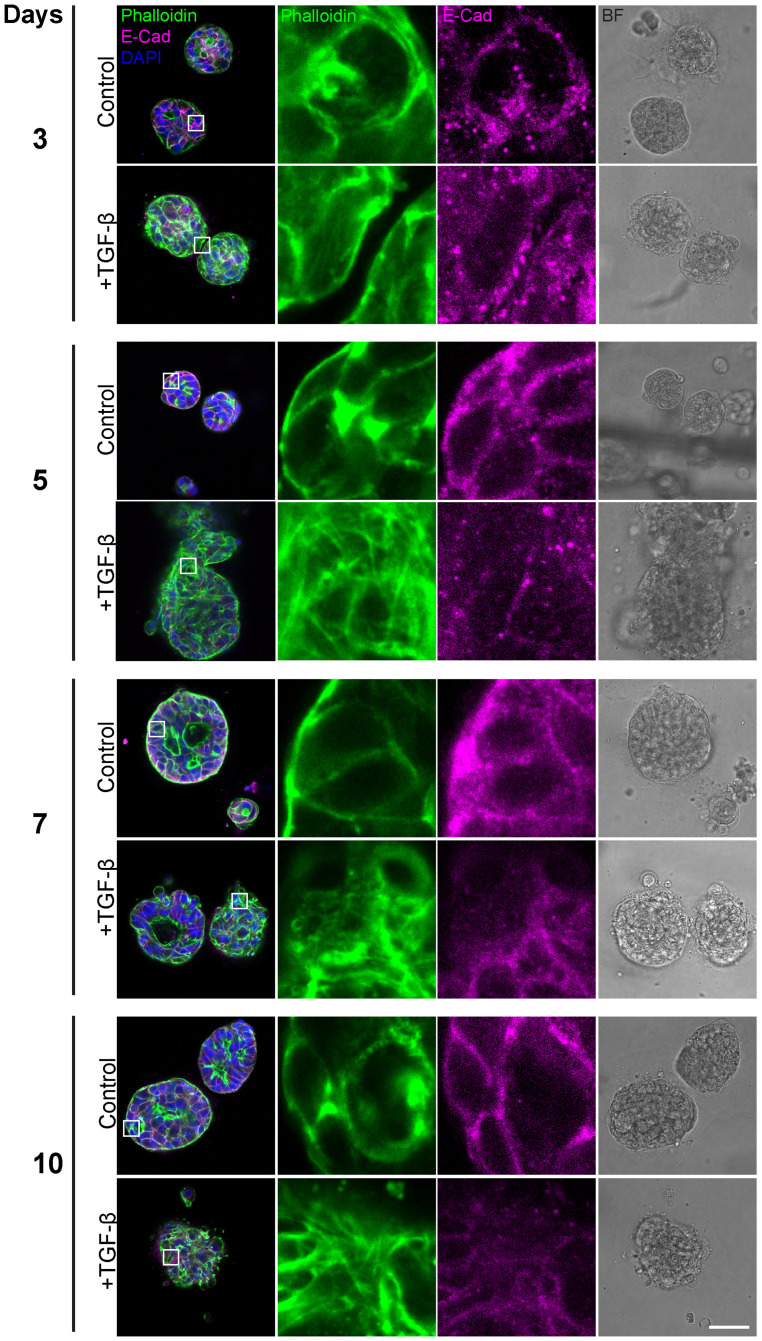
Gradual EMT progression in triple-positive PDOs. Patient-derived triple-positive (hormone receptor and HER2-positive) breast cancer organoids were cultured in two conditions: control (organoid culture medium) and TGF-β (EMT-medium). Organoids were analyzed over a time course of 3, 5, 7, and 10 days. Immunolabeling was performed with antibody for the epithelial marker E-Cadherin and counter-stained with DAPI to label cell nuclei (blue) and Phalloidin to label F-actin. Confocal imaging was used to capture detailed cellular changes along with bright field images (right column). Representative images from each time point and condition are shown. Enlargement of the squared areas highlight actin rearrangement into stress fibers and E-Cadherin downregulation in the TGF-β-treated group. Bar= 50µm.

Having observed general morphological changes in the histological sections upon 10-day TGF-β induction, we sought to gain a more detailed understanding of these morphological phenotypes. To achieve this, we conducted additional experiments using immunofluorescence and confocal imaging techniques, including time-course analyses to capture the temporal progression of this process. First, we conducted a time course study exposing triple-positive PDOs to TGF-β (EMT-medium versus control) for 3, 5, 7, and 10 days (**Figure 2**). The results revealed EMT-like changes including cell elongation and stress fibers formation on day 3 of TGF-β induction. Over time, an increasing number of cells underwent morphological changes, ultimately leading to a uniform and homogeneous response to EMT induction including downregulation of E-Cadherin and the reorganization of cortical actin into stress fibers, accompanied by loss of luminal structure and overall architectural changes.

**Figure 3:**
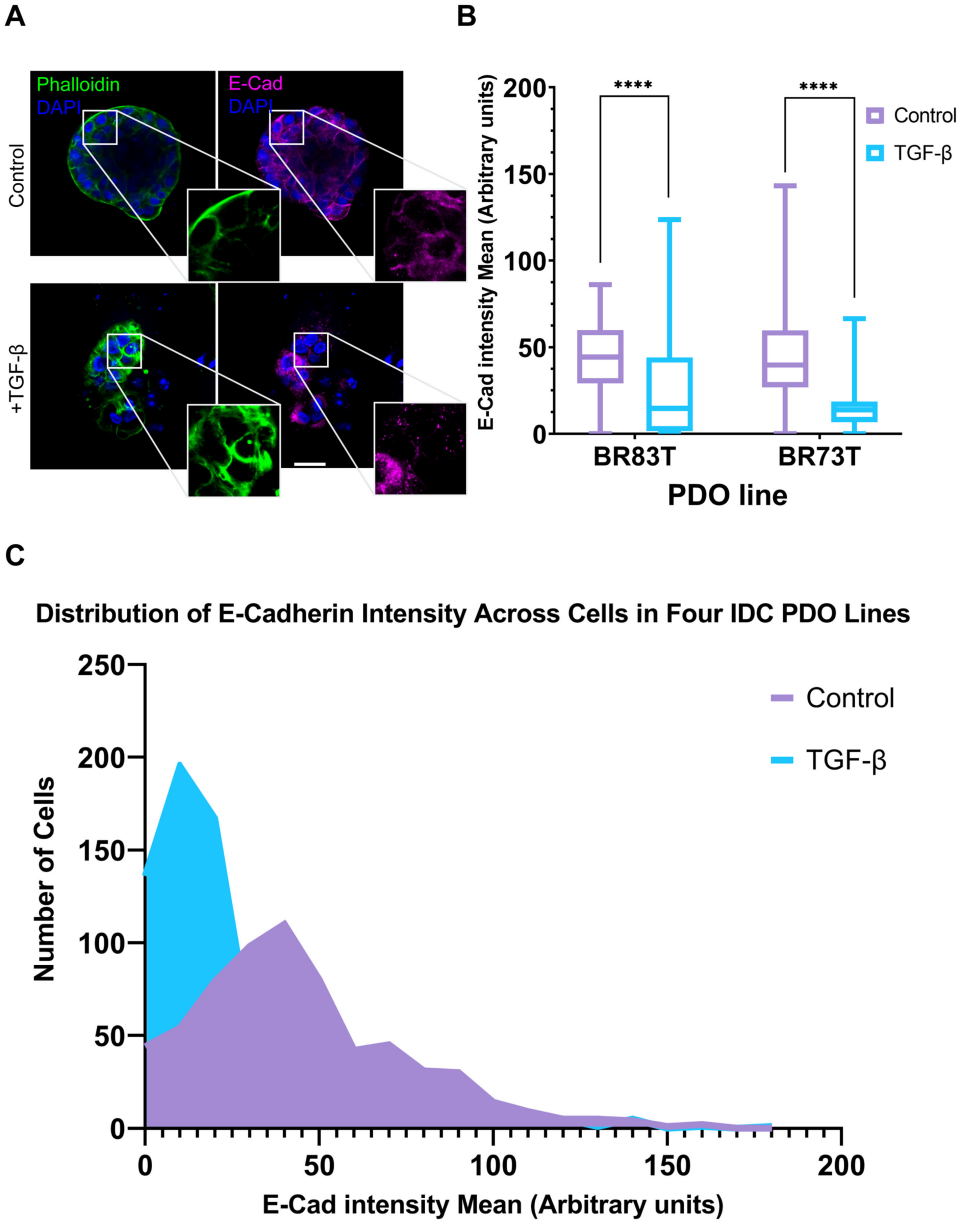
Modeling and quantification of EMT in breast cancer PDOs. **(A)** Control and TGF-β treated (EMT-medium) patient-derived hormone-receptor positive breast cancer organoids were cultured for 8 days. The organoids were then immunolabeled with an antibody for the epithelial marker E-Cadherin (magenta) and counter-stained with DAPI to label cell nuclei (blue) and Phalloidin to label F-actin (green). Confocal imaging was used to capture detailed cellular changes. Enlargement of the squared areas highlights actin rearrangement as stress fibers and E-Cadherin downregulation in the TGF-β treated organoids. Bar = 50 µm. **(B)** E-Cadherin mean intensity measurements in individual cells was measured following cell segmentation using CellPose3, a deep neural network-based instance segmentation algorithm (44).The algorithm supports designating different channels as the nucleus and cytoplasm in order to facilitate better segmentation. Here, DAPI was used as the nucleus marker and Phalloidin as the cytoplasm marker. E-Cadherin intensity was measured in cells from two hormone receptor-positive breast cancer patient-derived organoids (PDOs), BR73T (n=386) and BR83T (n=50). An unpaired t-test was performed to compare the mean E-Cadherin intensities between control and TGF-β treated PDOs. Data are presented as mean ± SEM. p values ****<0.0001. **(C)** Distribution of E-Cadherin intensity (x-axis) in cells from four breast cancer PDOs with and without EMT induction using TGF-β as described in **(B)**. The y-axis represents the number of cells.

Based on these findings, we proceeded to investigate the susceptibility of PDO lines derived from different breast cancer subtypes to EMT induction. The HR+ PDOs were subjected to EMT induction using TGF-β (EMT-medium versus control). Confocal imaging of organoids, stained for F-actin with Phalloidin and immunolabeled for E-Cadherin, revealed similar alterations following induction (**Supplementary Figure 2**; **Figure 3A**). Notably, PDOs demonstrated a uniform, homogeneous response pattern to TGF-β, which was comparable to the response observed in 7- and 10-day exposure of the triple-positive IDC PDOs described in the previous section.

To evaluate the observed phenotypic changes in the confocal microscopy images, we utilized the CellPose3 segmentation algorithm (44). This approach allowed us to measure and quantify E-Cadherin down-regulation by assessing the mean E-Cadherin intensity for each cell. A significant reduction in E-Cadherin levels was observed following EMT induction (**Figure 3B**). E-Cadherin down-regulation was consistently noted across all four IDC PDO lines, as shown in **Figure 3C**. It is noteworthy that the CellPose3 algorithm, initially trained for single-cell segmentation across various tissues, has not been specifically tailored for PDOs, especially those exhibiting altered morphology. We validated the segmentation outcomes by comparing them with manual segmentation and by evaluating the similarity in nuclear area between control and treatment groups, yielding similar results (**Supplementary Figure 2**; **Supplementary Table 1**). The minor discrepancy between the algorithmic and manual segmentations may stem from changes in phalloidin staining in the TGF-β group, where, due to EMT-induction it is not always confined to cell borders, causing some cells in the treatment group to be omitted. This discrepancy, however, reinforces our findings, as it likely excludes cells with E-Cadherin downregulation.

**Figure 4:**
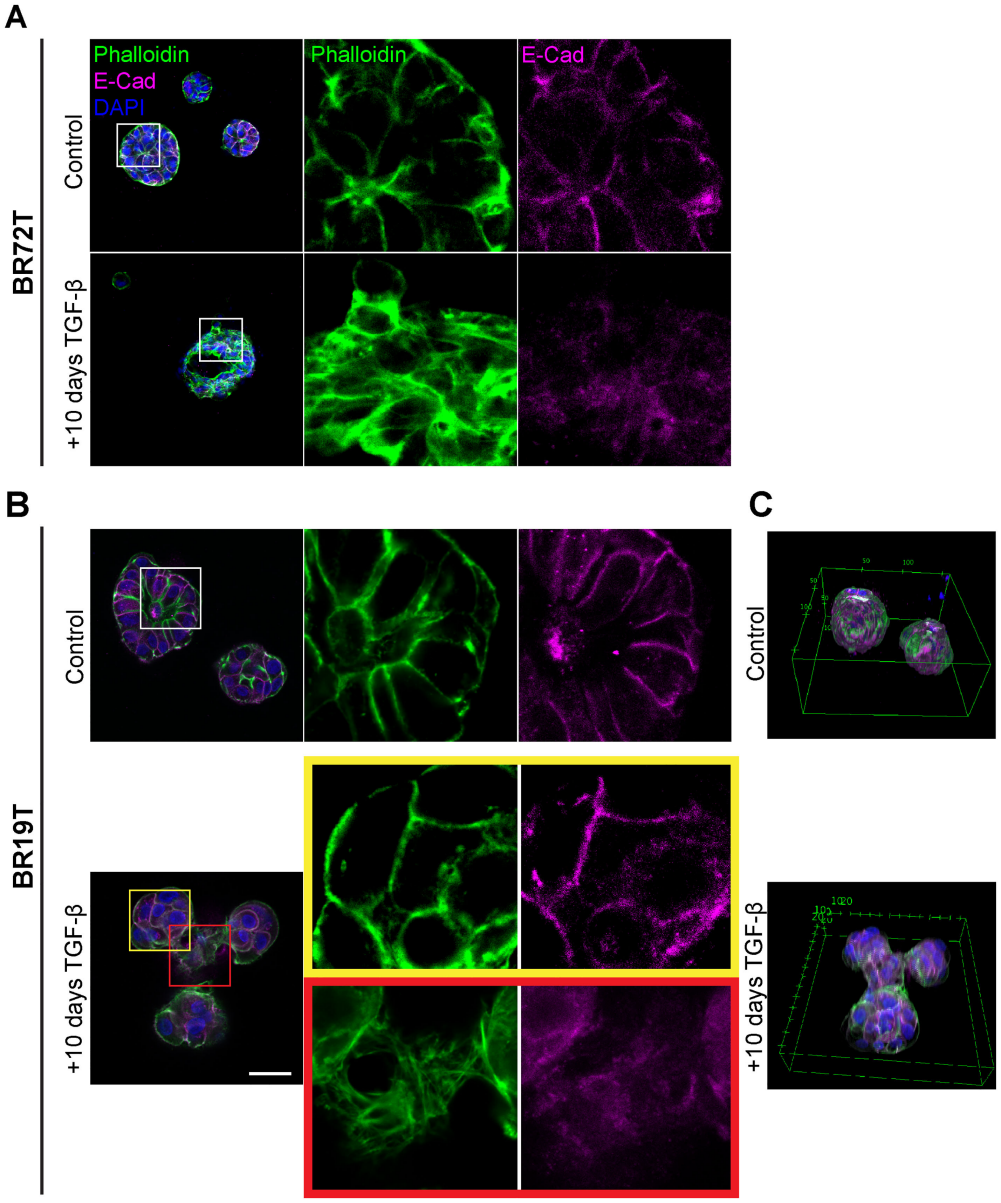
Heterogenous EMT phenotypes correlate with tumor histology. Control and TGF-β treated (EMT-medium) patient-derived hormone-receptor positive breast cancer organoids were cultured for 10 days. The organoids were then immunolabeled with an antibody for the epithelial marker E-Cadherin (magenta) and counter-stained and counter-stained with DAPI to label cell nuclei (blue) and Phalloidin to label F-actin (green). Confocal imaging was used to capture detailed cellular changes. The images show representative images of control organoids (top) versus TGF-β treated organoids (bottom). **(A)** Hormone receptor-positive IDC organoids (BR72T) exhibit a uniform susceptibility pattern, with most cells within the organoid responding to TGF-β. Enlargement of the squared area reveals actin rearrangement into stress fibers and downregulation of E-Cadherin in the TGF-β-treated group. **(B)** Mucinous carcinoma organoids (BR19T) show a selective, heterogeneous susceptibility pattern, where only a specific subset of cells responds to TGF-β. Enlargement of the yellow squared area highlights cells that remain unchanged following TGF-β treatment, while enlargement of the red squared area illustrates actin rearrangement into stress fibers and E-Cadherin downregulation in the responding cells. Bar= 50µm. **(C)** 3D reconstruction of Mucinous carcinoma organoids demonstrates the changes in organoids structure and organoids fusion upon treatment in cancerous organoids, formed by EMT-induced cells.

The global phenotypic cellular EMT-like changes in HR+ and triple positive IDC PDOs following TGF-β induction protocol (EMT-medium) were consistent across all IDC PDOs (**Figures 2**, **3A**, **4A**; **Supplementary Figure 3**). However, PDOs derived from mucinous carcinoma exhibited a rather heterogeneous and selective response to TGF-β induction (**Figures 4B, C**), even at long exposure. These results demonstrate the potential of modeling EMT in PDOs to reveal heterogeneous cellular responses, and possibly reflect the contribution of cellular plasticity in tumor heterogeneity.

### 3.3 PDOs well-bottom adhesion reflecting EMT-induced cancer-cell migration and invasion.

**Figure 5:**
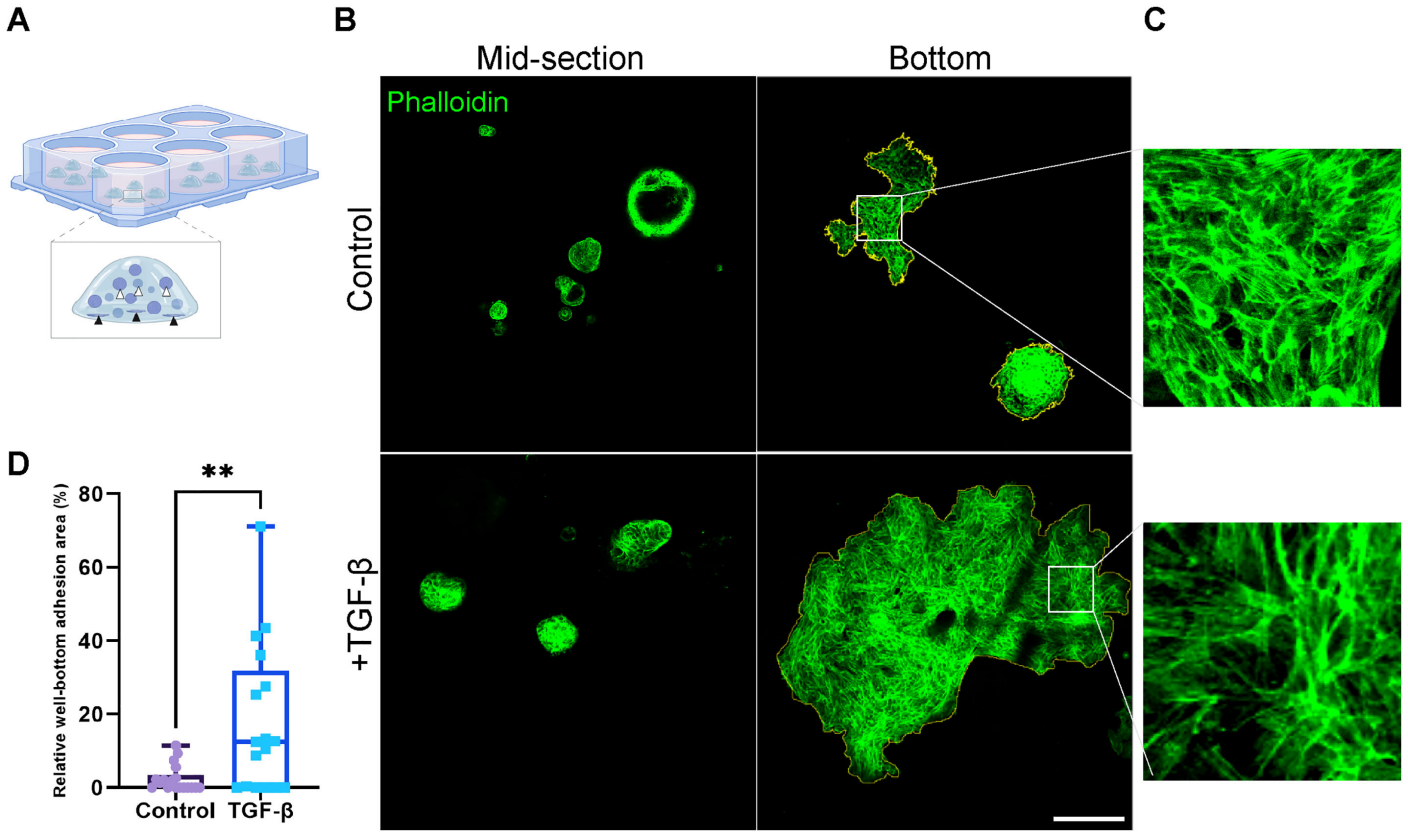
Area of well-bottom adhesion as an indicator for EMT-induced PDOs migration and invasion. **(A)** Illustration showing hormone receptor positive breast cancer organoids adhering to the well bottom. White arrows indicate typical organoids at the mid-section of the BME drop, while black arrows point to adherent organoids at the well bottom. **(B)** Confocal imaging of breast cancer organoids stained with Phalloidin to label F-actin (green). Top: control group. Bottom: organoids treated with TGF-β to induce EMT for 8 days. Left: organoids at the mid-section of the BME drop. Right: adherent organoids at the well bottom. The adhesion area on the right images was marked to calculate the relative well-bottom adhesion area (yellow) **(C)** Enlargement of the squared area shows actin rearrangement into stress fibers in adherent organoids in both groups. **(D)** Graphs show the relative well-bottom adhesion area in TGF-β treated group compared to the control group. **P-value = 0.0034. Unpaired T-test. n=18.

While organoids typically display a 3D structure throughout the BME drop, upon induction with TGF-β, we observed a marked increase in the adhesion of organoids to the well bottom (**Figures 5A, B**; **Supplementary Figure 4**). The adhesion observed indicates the migration and invasion of organoids within the BME, suggesting their ability to move through the extracellular matrix upon EMT induction resulting in organoids migration towards well bottom and adhesion. The difference in adherence pattern between control and EMT induced organoids was quantified by measuring the relative adhesion area on the well bottom: a larger relative adhesion area corresponds to greater migratory activity (**Figure 5D**). Curiously, both control and TGF-β-treated (EMT-medium) adherent organoids exhibited re-organization of actin into stress fibers and adopted a mesenchymal-like morphology (**Figures 5B, C**). Thus, suggesting that organoids’ attachment to stiff culture plate induces the formation of mesenchymal-like features highlighting the relevance of 3D models to study EMT in patient-derived cultures.

### 3.4 PDOs embody epithelial plasticity and heterogeneity

**Figure 6:**
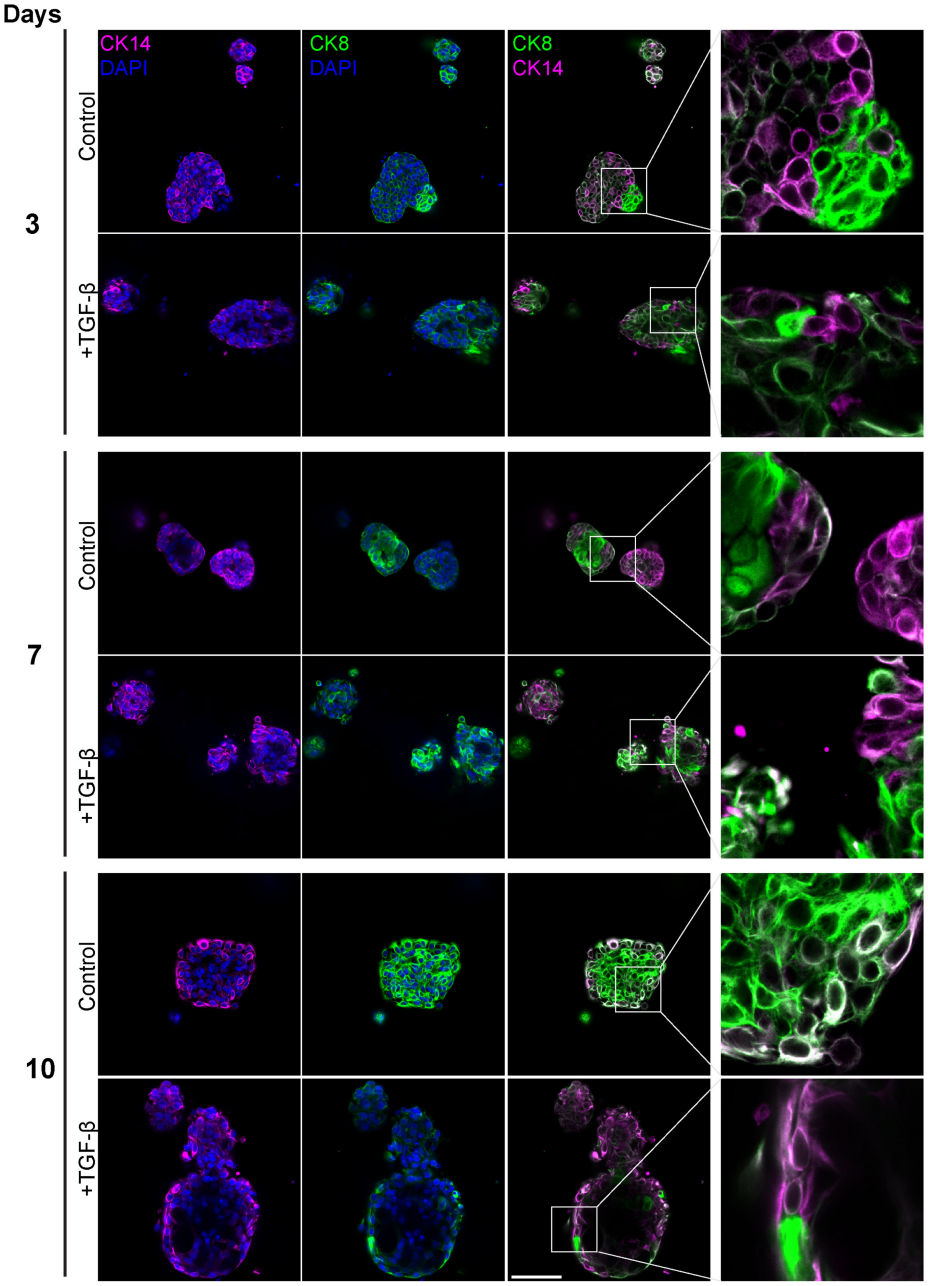
Altered luminal and myoepithelial distribution induced by TGF-β. Control and TGF-β treated (EMT-medium) patient-derived triple-positive (hormone receptor and HER2-positive) breast cancer organoids were cultured over a time course of 3, 7, and 10 days. Immunolabeling was performed with antibodies for CK8 to mark luminal cells (green) and with CK14 to label myoepithelial cells (magenta) and visualized using confocal microscopy. Top: Control. bottom: TGF-β induced organoids. Representative images from each time point and condition are shown, enlargement of the squared area highlights specific areas of heterogeneity, in which cells expressing different epithelial markers are in neighbor. Bar= 50µm.

To evaluate the contribution of distinct epithelial subpopulations to cell plasticity and tumor heterogeneity, PDOs were treated with TGF-β (EMT-medium) for 3, 7 and 10 days, and immunolabeled with antibodies for luminal cytokeratin 8 (CK8) and myoepithelial cytokeratin 14 (CK14) (**Figure 6**). The control samples demonstrated a gradual spatial organization of outer basal and inner luminal layers, demonstrating the described PDO model’s capability to recapitulate the original tissue’s architecture and to organize over time. Of note, unlike PDOs from normal tissue that demonstrate organoid architecture resembling normal tissue, cancer organoids can exhibit diverse morphological structures depending on tumor grade and subtype (36). On day 3 of the time-course experiment, control organoids showed no specific spatial organization of CK8 and CK14 expressing cells. However, by later time points, control organoids developed a distinct structure with a majority of luminal cells and a thin layer of CK14-positive cells at the outer border. In contrast, the TGF-β-treated organoids did not exhibit this spatial organization. Instead, they displayed disrupted and disorganized cellular architecture at all time points, demonstrating the impact of TGF-β on the structural integrity of the PDOs.

Furthermore, a notable decrease in the luminal to basal cell ratio after 10 days of TGF-β treatment, suggesting that EMT increased the prevalence of basal-like aggressive cells at the expense of luminal cells. These results demonstrate the capacity of PDOs to replicate the spatial and functional dynamics of the original tumor.

## 4 Discussion

Fundamental to tumor progression, metastasis and therapy resistance, cellular plasticity holds a pivotal role. The inherent complexity of plasticity complicates the development of effective models. In this study, we demonstrated the possibility to model EMT in breast cancer PDOs, enabling the assessment of cell plasticity in a human-relevant context. Intriguingly, PDOs preserved the original tissue architecture and displayed epithelial plasticity and invasiveness, demonstrated by EMT features, epithelial cell type alterations, and movement within the extracellular matrix.

This study underscores the substantial potential of PDOs in advancing our understanding of cancer cell plasticity. Complementary to existing breast cancer models, PDOs accurately preserve the complexity of human tissue, maintaining the inter-tumor and inter-patient heterogeneity that is often not represented in cell lines and spheroids cultures. Additionally, PDOs enable the study of dynamic biological processes that cannot be captured by static, fixated tissue samples (36, 39, 49–51).

EMT in cancer allows cancer cells to invade and migrate, thus contributing to cancer progression. The here established well-bottom adherence quantification essay is used as a correlative measurement to assess organoids’ capability to invade extracellular matrix (BME) and migrate towards well-bottom. Evaluating adherent organoids in the control samples highlighted the importance of utilizing a 3D model system for investigating cell type transition processes, as the adherence to a monolayer significantly influences cell identity and morphology.

Microscopy-based analysis facilitated the establishment of a robust methodology for evaluating EMT susceptibility in primary PDO cultures. Microscopy offers significant advantages in this context by providing rapid, high-resolution spatial context of cellular and subcellular features within intact organoid structures. Microscopy-based analysis is feasible also if only a small number of organoids is available, without going through extensive culture expansions and passaging. Complementary single-cell analysis methods are possible but have the disadvantage of significant cell loss and stress associated with organoid dissociation in single-cell methods, which can affect EMT outcomes and introduce bias. Microscopy is particularly beneficial for “primary” (early-passage) PDO cultures, where preserving the original genomic characteristics and tumor heterogeneity is essential. This advantage is underscored by the fact that expanding PDOs is often impractical and may lead to alterations in their genomic profiles and clonal diversity over time (52–54). By preserving the three-dimensional structure and cellular interactions, microscopy provides a comprehensive view of EMT processes, offering insights that might be missed with dissociative single-cell methods, and ensuring that genomic and functional analyses remain aligned.

A patient-derived model facilitates the exploration of correlations between histopathological characteristics and phenotypes and outcomes *in-vitro*. Although definitive conclusions necessitate larger cohorts, several noteworthy observations emerged. Notably, we identified a correlation between HR+, intermediate-risk IDC in premenopausal women and a uniform response to EMT. Additionally, PDOs of mucinous carcinoma demonstrated a selective susceptibility to EMT, even upon prolonged exposure to TGF-β. This observation might align with the typically less aggressive behavior of mucinous carcinoma compared to other breast cancer types.

Although PDOs offer a sophisticated model for studying cancer, they come with several limitations that must be considered for future research. These include the limited range of tissue types that can be successfully cultured as organoids, incomplete representation of the tumor microenvironment (such as interactions with immune cells and vasculature), and the variability in culture conditions. Additionally, the process of developing and maintaining PDOs can be resource-intensive and time-consuming.

Overall, the study underscores the unique value of PDOs as an effective model for investigating EMT. Microscopy-based image analysis enabled quantification of EMT induction in PDOs while preserving spatial architecture and tumor heterogeneity. Thus, PDOs offer a platform for elucidating the dynamics of EMT and reveal primary breast cancer susceptibility to cell plasticity. Future research would benefit from integrating spatial transcriptomics at the single-cell level to further decipher cancer cell plasticity and provide more detailed, quantitative insights. By providing deeper insights into cancer cell plasticity, PDOs could facilitate the development of novel therapeutic approaches aimed at modulating this plasticity to achieve more favorable clinical outcomes.

## Data Availability

The raw data supporting the conclusions of this article will be made available by the authors, without undue reservation. All codes related to E-Cadherin mean intensity measurements can be found at https://github.com/amirza1/ECadMeanOrganoids. This Python library will also include our code to call Cellpose to perform segmentation.

## Supplementary material

Supplementary Figure 1 H&E Staining Illustrates Histological Features of Triple-Positive IDC Tissue with Matched Control and TGF-β-treated PDOs. Hematoxylin and Eosin (H&E) staining was performed on the original tissue of patient-derived organoids (PDO) BR73T, untreated control PDO BR73T, and 10 days TGF-β-induced PDO BR73T. Control and treated PDOs were fixed, embedded in paraffin to create Formalin-Fixed Paraffin-Embedded (FFPE) blocks, and then sectioned onto slides for H&E staining. Bar = 50µm.

Supplementary Figure 2 Cell segmentation and validation in organoid confocal microscopy images. Organoid image segmentation into individual cells was performed using CellPose3. The segmentation was defined with the nucleus assigned to the blue channel (DAPI) and the cytoplasm to the green channel (Phalloidin), generating an instance segmentation map of the cells, as illustrated in **(A)**. Validation of the segmentation was conducted by analyzing the distribution of the nucleus area in each cell, confirming that the segmentation was consistent between Control and TGF-β groups, as shown in **(B)**.

Supplementary Figure 3 EMT characterization in an additional hormone-receptor positive IDC PDO line. Control and TGF-β treated (EMT-medium) patient-derived hormone-receptor positive breast cancer organoids (BR63T) were cultured for 8 days. The organoids were then immunolabeled with an antibody for the epithelial marker E-Cadherin (magenta) and counter-stained with DAPI to label cell nuclei (blue) and Phalloidin to label F-actin (green). Confocal imaging was used to capture detailed cellular changes. The images show control organoids (top) versus TGF-β treated organoids (bottom). Enlargement of the squared areas highlights actin rearrangement as stress fibers and E-Cadherin downregulation in the TGF-β treated organoids. Bar= 50µm.

Supplementary Figure 4 PDOs adhering to the well bottom. Two lines of hormone-receptor positive breast cancer organoids were cultured (control and TGF-β treated) for 8 days. Confocal imaging of organoids adhering to the bottom of the well, immunolabeled with Phalloidin to label F-actin (green) are shown. Left: control. Right: TGF-β treated organoids. Bar = 500µm.
