# Supplementary figures and images for: Modeling epithelial-mesenchymal transition in patient-derived breast cancer organoids
Source: Front Oncol. 2024 Oct 14;14:1470379. doi: 10.3389/fonc.2024.1470379 (PMC11513879; doi:10.3389/fonc.2024.1470379)

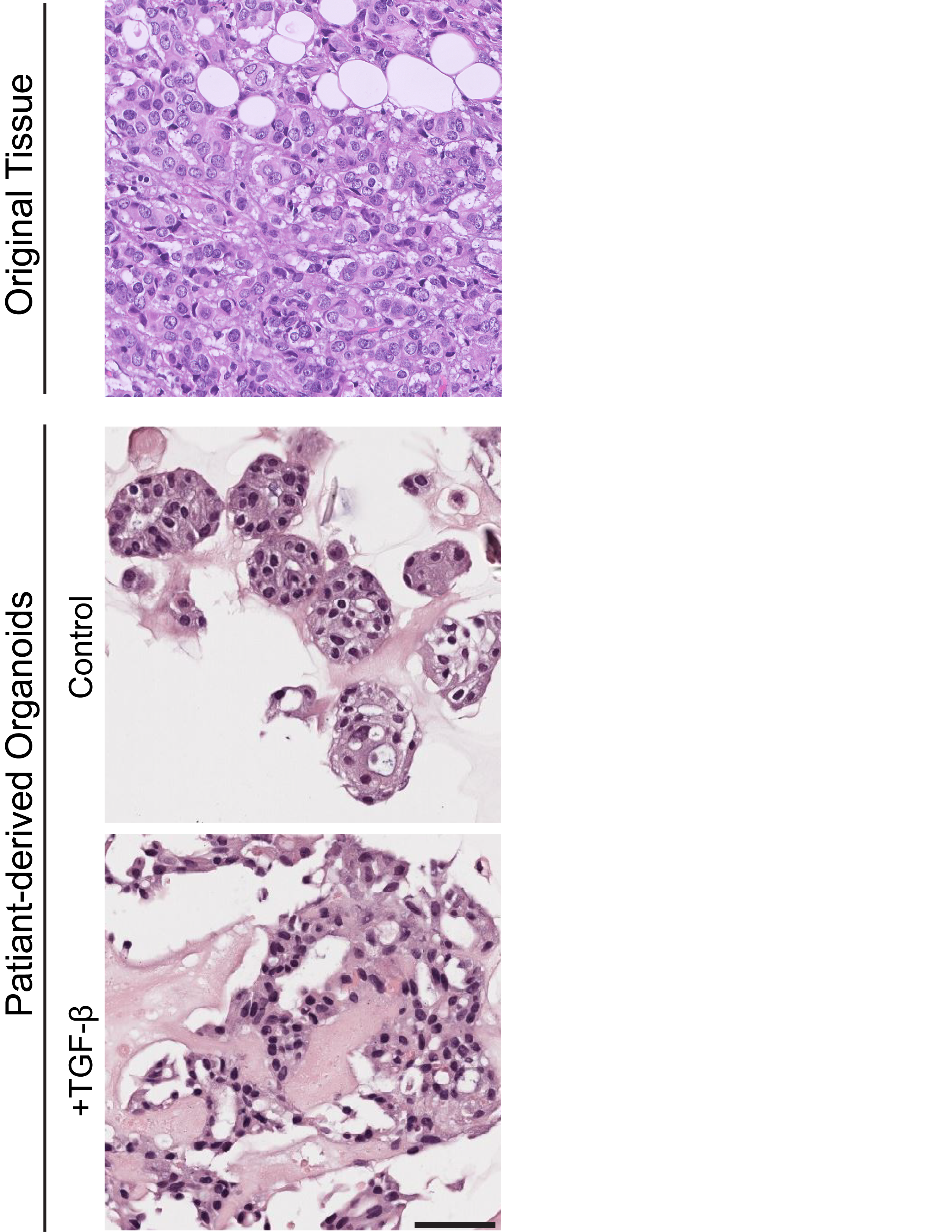

Supplement: Supplementary Figure 1 — H&E Staining Illustrates Histological Features of Triple-Positive IDC Tissue with Matched Control and TGF-β-treated PDOs. Hematoxylin and Eosin (H&E) staining was performed on the original tissue of patient-derived organoids (PDO) BR73T, untreated control PDO BR73T, and 10 days TGF-β-induced PDO BR73T. Control and treated PDOs were fixed, embedded in paraffin to create Formalin-Fixed Paraffin-Embedded (FFPE) blocks, and then sectioned onto slides for H&E staining. Bar = 50µm. [file DataSheet1.zip › Supplementary Figure 1 .tif]

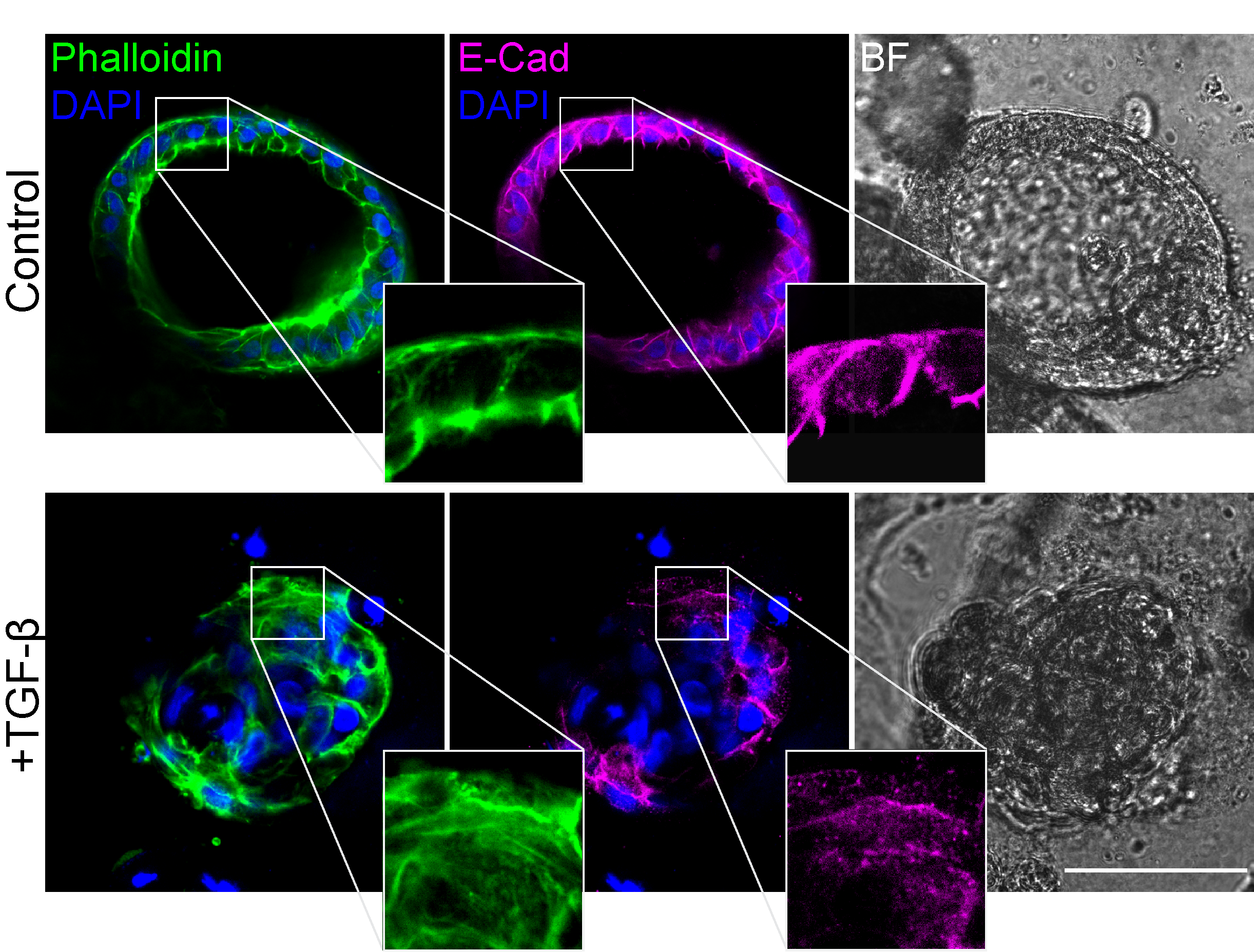

Supplement: Supplementary Figure 1 — H&E Staining Illustrates Histological Features of Triple-Positive IDC Tissue with Matched Control and TGF-β-treated PDOs. Hematoxylin and Eosin (H&E) staining was performed on the original tissue of patient-derived organoids (PDO) BR73T, untreated control PDO BR73T, and 10 days TGF-β-induced PDO BR73T. Control and treated PDOs were fixed, embedded in paraffin to create Formalin-Fixed Paraffin-Embedded (FFPE) blocks, and then sectioned onto slides for H&E staining. Bar = 50µm. [file DataSheet1.zip › Supplementary Figure 2.tif]

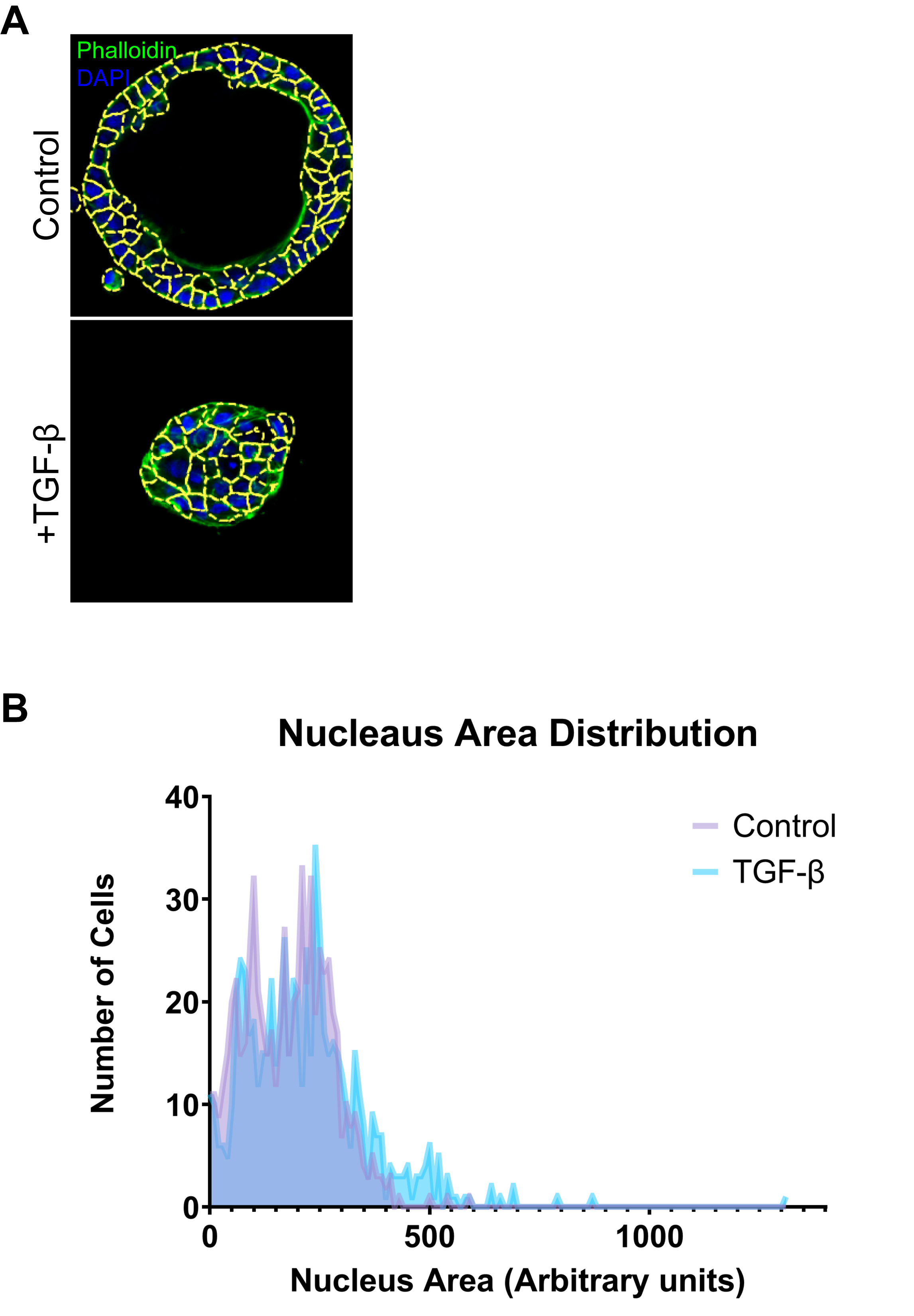

Supplement: Supplementary Figure 1 — H&E Staining Illustrates Histological Features of Triple-Positive IDC Tissue with Matched Control and TGF-β-treated PDOs. Hematoxylin and Eosin (H&E) staining was performed on the original tissue of patient-derived organoids (PDO) BR73T, untreated control PDO BR73T, and 10 days TGF-β-induced PDO BR73T. Control and treated PDOs were fixed, embedded in paraffin to create Formalin-Fixed Paraffin-Embedded (FFPE) blocks, and then sectioned onto slides for H&E staining. Bar = 50µm. [file DataSheet1.zip › Supplementary Figure 3.tif]

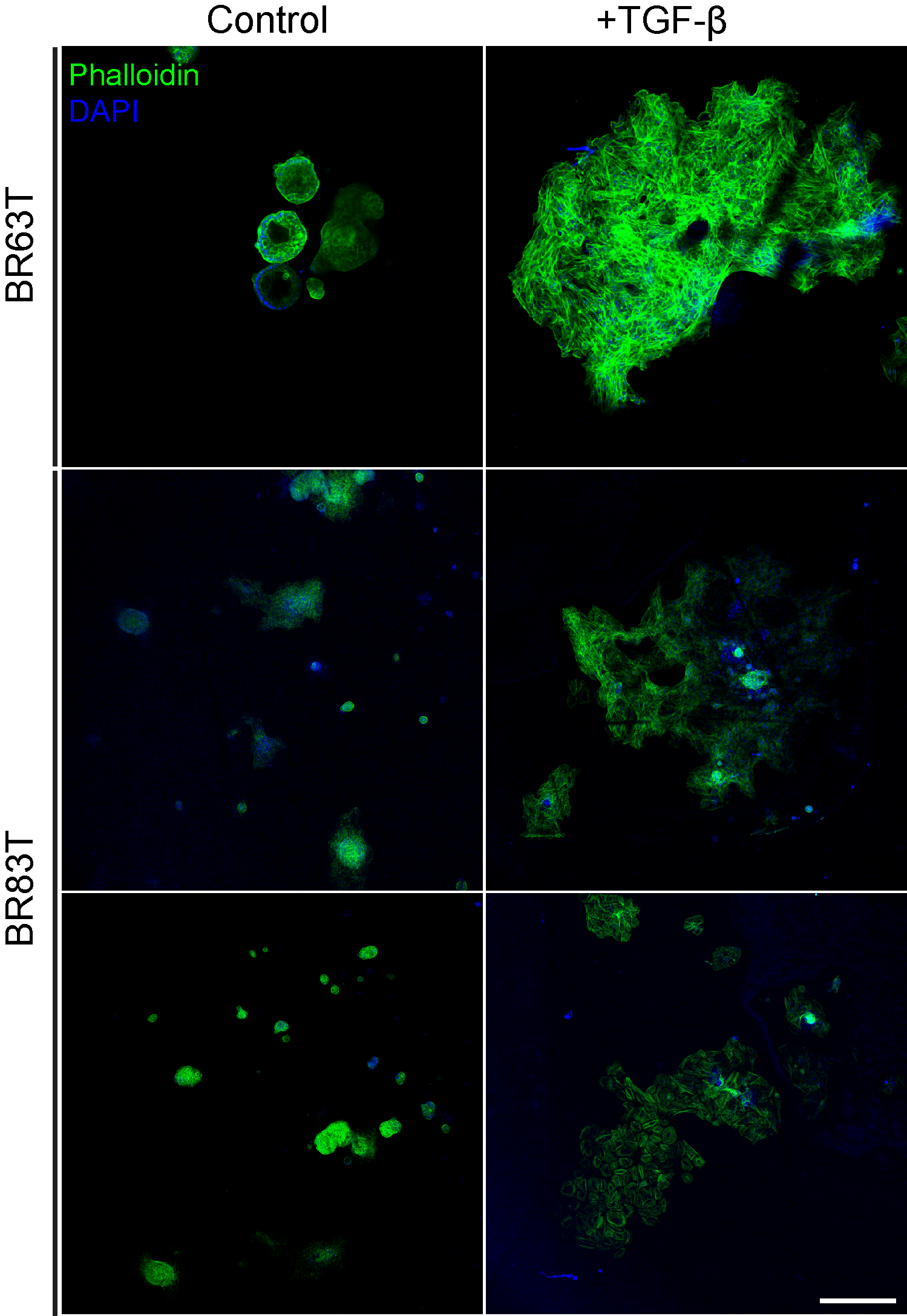

Supplement: Supplementary Figure 1 — H&E Staining Illustrates Histological Features of Triple-Positive IDC Tissue with Matched Control and TGF-β-treated PDOs. Hematoxylin and Eosin (H&E) staining was performed on the original tissue of patient-derived organoids (PDO) BR73T, untreated control PDO BR73T, and 10 days TGF-β-induced PDO BR73T. Control and treated PDOs were fixed, embedded in paraffin to create Formalin-Fixed Paraffin-Embedded (FFPE) blocks, and then sectioned onto slides for H&E staining. Bar = 50µm. [file DataSheet1.zip › Supplementary Figure 4.tif]
